# Transfer of microRNAs by extracellular membrane microvesicles: a nascent crosstalk model in tumor pathogenesis, especially tumor cell-microenvironment interactions

**DOI:** 10.1186/s13045-015-0111-y

**Published:** 2015-02-22

**Authors:** Li Zhang, C Alexander Valencia, Biao Dong, Meng Chen, Pu-Jun Guan, Ling Pan

**Affiliations:** Department of Hematology, West China Hospital, Sichuan University, Chengdu, 610041 China; Division of Human Genetics, Cincinnati Children’s Hospital Medical Center, Cincinnati, Ohio 45229 USA; Department of Pediatrics, University of Cincinnati College of Medicine, Cincinnati, Ohio 45229 USA; State Key Laboratory of Biotherapy, West China Hospital, Sichuan University, Chengdu, 610041 China

**Keywords:** Microvesicle, Exosome, MicroRNA, miRNA, Tumor, Microenvironment

## Abstract

Anticancer treatments aiming at killing malignant cells have been applied for decades but have been unsuccessful at curing the disease. The modern concept of tumor microenvironment, especially angiogenesis, suggests that the tumor is not only composed of malignant cells, but also consists of other groups of cells that work together. Recently, genetic message transfer has been revealed between tumor cells and their microenvironment. The latest cell-derived vector, extracellular membrane microvesicles (EMVs), has been found to provide membrane protection and allowed to deliver genetic information beyond the cells. Additionally, EMV-associated microRNAs are involved in a variety of cellular pathways for tumor initiation and progression. Previous published reviews have focused on miRNA that included EMVs as a sensitive marker for tumor monitoring in clinical applications that are based on the alteration of their expression levels in conjunction with disease occurrence and progression. From the aspect of cellular crosstalk, this article will review the role of EMV-mediated microRNA transfer in tumor pathogenesis, including tumor treatment obstacles, history and features, and current research in inflammatory/immune pathologies, as well as in solid tumors and hematological malignancies. This nascent crosstalk model will provide a novel insight into complementing the classic mechanisms of intercellular communication and contribute to the potential therapeutic strategy via small RNA molecule-carrying EMVs for multimodality treatment of cancer.

## Introduction

Cancer is a leading cause of death in developed nations and a growing worldwide epidemic. Previous understanding of tumor pathogenesis mainly focused on the tumor itself, including the identification of oncogenes and suppressor genes. Accordingly, current anticancer therapies are mainly by means of surgery, radiation, and chemotherapy, which directly target the malignant compartment. However, cancer, the majority of which is still incurable, has evolved to be one of the most remarkable global problems. There are unexpected obstacles which prevent conventional treatments by using tumor-targeted toxins and resection to be curative. Thereby, much effort has been made in other aspects on exploring tumor pathogenesis in depth. The latest perspectives in oncology have begun to change from autonomous mechanisms of malignant cells to crosstalks with other nonmalignant adjacent components. A new concept, tumor microenvironment, is being placed forward to explain the reciprocal causation between tumors and their surrounding components, and this may potentially overcome the existing therapeutic limitations.

Tumor microenvironment was firstly described by Judah Folkman in 1971 [1] and has been recognized as one of the major hallmarks of cancer. Referring to the *National Cancer Institute* (*NCI*) *Dictionary of Cancer Terms*, a tumor microenvironment represents a well-orchestrated integration where the tumor exists and is constituted by surrounding blood vessels, immune cells, fibroblasts, other cells, signaling molecules, and the extracellular matrix. This concept was based on the ‘seed and soil’ hypothesis to explain the non-random pattern of metastasis by the English surgeon Stephen Paget in 1889. Then, it was presented to describe tumor cell-host cell interactions for the first time by the American scholar Lord EM, et al. in the 1970s [2]. These encouraging studies on tumor microenvironment led to new perspectives for anticancer therapy [3] and paved the way to the birth of thalidomide, the first-generation immunomodulatory drug, into the antitumor regimen in the early years of the past century. Since then, many efforts have been made to design novel therapeutic drugs and conduct clinical trials, aiming to influence the ongoing pro-tumor environment.

The role of angiogenesis in the tumor microenvironment has always retained an essential focus in preclinical medical research on tumor progression. It was presented by Willis in 1948 and originated from the hypothesis of angiogenesis-dependent tumor, as tumor cells were observed to acquire a new phenotype to participate in the formation of blood channels [4]. Thereafter, it has inspired new investigations to clarify the mechanism of angiogenesis. To date, pro-tumor angiogenesis has been developed as a pivotal therapeutic target in the clinical setting, especially for lymphoproliferative disease, such as multiple myeloma (MM) and aggressive lymphoma. Sprouting formation and splitting growth are two primary ways of angiogenesis development in which both are initiated from the preexisting vasculature. This is different from angioblast-mediated vasculogenesis. Angiogenesis and vasculogenesis potentially play distinct roles and may be the center part in the etiology of primary and recurrent neoplasms, respectively [5]. Although blockade in both angiogenic and vasculogenic pathways are attractive theoretical targets, in clinic work, only the first and second generation of anti-angiogenic therapies have been widely used as one of the most rapidly developing aspects of anticancer treatment, to some extent, because a series of questions concerning the role of vasculogenesis still remains unanswered. Additionally, a mutated mouse model with defective angiogenesis presented its resistance to transplanted tumors [6]. This suggested that angiogenesis may be involved in tumor growth with more power than vasculogenesis. Hence, the studies of tumor angiogenesis have continuously attracted more attention in tumorigenesis, which waits for a better understanding of the interaction between malignant cells and epithelial cells in the microenvironment.

The discovery of the tumor microenvironment, angiogenesis, raises an important question on the interplay between cancer cells and their neighboring components; how do cancer cells deliver messages to the remaining normal endothelial capillaries? In addition to that, the neovasculogenesis, the tumor microenvironment, mainly harbors genetic arrangement abnormalities. Significantly, the consensus tumor-specific genetic aberrations were found in both tumor cells and the corresponding microvascular endothelial cells [7-9]. Two classic models of cellular mutual communication, namely, the direct cell-to-cell contact and the membrane receptors with ligands, allow comprehending the possibility of tumor-induced endothelial-cell growth in the microenvironment. However, taking into consideration the wide spread of extracellular nucleases, responsible for the rapid clearance of extracellular secretion of nucleic acid fragments, both models are inappropriate methods to explain why the neoplastic epithelium exhibits specific genetic abnormalities or whether any special carriers for transferring nucleic acids into the cells already existed within the tumor niche or just evolved from the distant regions. This review will focus on discussing novel mechanisms that trigger and influence the tumor and microenvironment crosstalk.

### Extracellular membrane microvesicles, mainly aggregating and selectively including miRNAs, are uncovered as a nascent crosstalk model for cellular communication

#### History and concept of EMVs

Extracellular membrane microvesicles (EMVs), new performers participating in microenvironment conformation, are circular fragments of membrane released from the endosomal compartments as exosomes with diameters of 30 to 100 nm or shedding from the surface membranes of most cell types as microvesicles with diameters of 50 to 2,000 nm [10]. Recently, EMVs are encouragingly observed to carry and release deoxyribonucleic acid (DNA), message ribose nucleic acid (mRNA), microRNAs (miRNAs), and proteins to target locations or associated cells [11-13]. The original concept of EMVs dates back to 1946 when Chargaff and West hypothesized that cell-free plasma may contain a subcellular factor promoting the clotting of blood [14]. Twenty years later, microvesicles were observed under the electron microscope which were derived from platelets [15]. The other type of EMVs, exosomes, was found by Johnstone et al. from sheep reticulocytes in 1987 [16]. These studies established a foundation for the latest findings which discovered EMVs in bodily fluids [17], namely, blood, urine, and bile [18-20], and more recently in the tumor microenvironment. Until now, differential ultracentrifugation, the golden standard method for separating and purifying EMVs, is incapable of distinguishing between exosomes and microvesicles [21]. In contrast, commercial kits are based on the principle of aqueous gradient solubility differences between various lipids and nanoparticles to capture EMVs and then use either 0.2-micrometer (μm) pore size filters or artificially synthesized molecular sieves to enrich exosomes. In this review, we describe EMVs that include both exosomes and microvesicles because they cannot be precisely separated by current methods.

### miRNAs selectively encoded into EMVs

As a novel class of regulators, miRNAs are the core elements in EMVs. Sequence analysis showed that there was a diverse collection of the exosomal RNA species among which miRNAs were the most abundant, making up over 76% of all mappable reads [22]. miRNAs are endogenous approximately 22 nt RNAs that play important gene regulatory roles to specify mRNA cleavage or repression of translation by pairing to the messages of protein-coding genes [23]. In total, 2,588 mature human miRNAs have been registered at the miRBase 21.0 and are predicted to target more than 5,300 human genes, which represented 30% of our exome [24], and the miRNA-mRNA regulatory network reflects extremely subtle combinatorics, both in terms of target multiplicity (more than one target per miRNA) and signal integration (more than one miRNA per target gene) [25]. Scientists have identified that vesicles which were released from both the human and murine mast cell lines contain approximately 121 miRNA molecules [26]. Moreover, miRNA expression has been defined in circulating plasma microvesicles of normal subjects [27]. Recent lines of evidence have revealed that miRNA exchange between cells may be accomplished through microvesicles [26]. In addition, other studies showed that the specific miRNAs are primarily found extracellularly, and those may change depending on physiological conditions [28,29]. It signifies that miRNA may be transported to the extracellular compartment by being selectively packed into the EMVs. Several studies have proposed models to explain whether specific signaling pathways exist to modify transport and packing of EMVs. Studies have reported that the secretion of exosomes may be triggered by the ceramide and neutral sphingomyelinase, but not by the endosomal sorting complex as previously thought [30,31]. Specifically, purified exosomes were observed to be enriched in ceramide and reduction of exosome release resulted from neutral sphingomyelinases inhibition [30,31]. We are just beginning to understand the mechanisms of EMVs’ regulation, but the underlying molecular mechanisms involving microRNAs being in EMV have yet to be elucidated. Occurrence of EMV-mediated miRNA transfer has been indirectly confirmed by detecting the altered expression levels of internal miRNAs in both donor and recipient cells. In the future, fluorescence signal amplification by a confocal imaging system may allow us to directly study EMV transfer.

### Characteristics of EMV-mediated miRNA transfer

**Figure 1:**
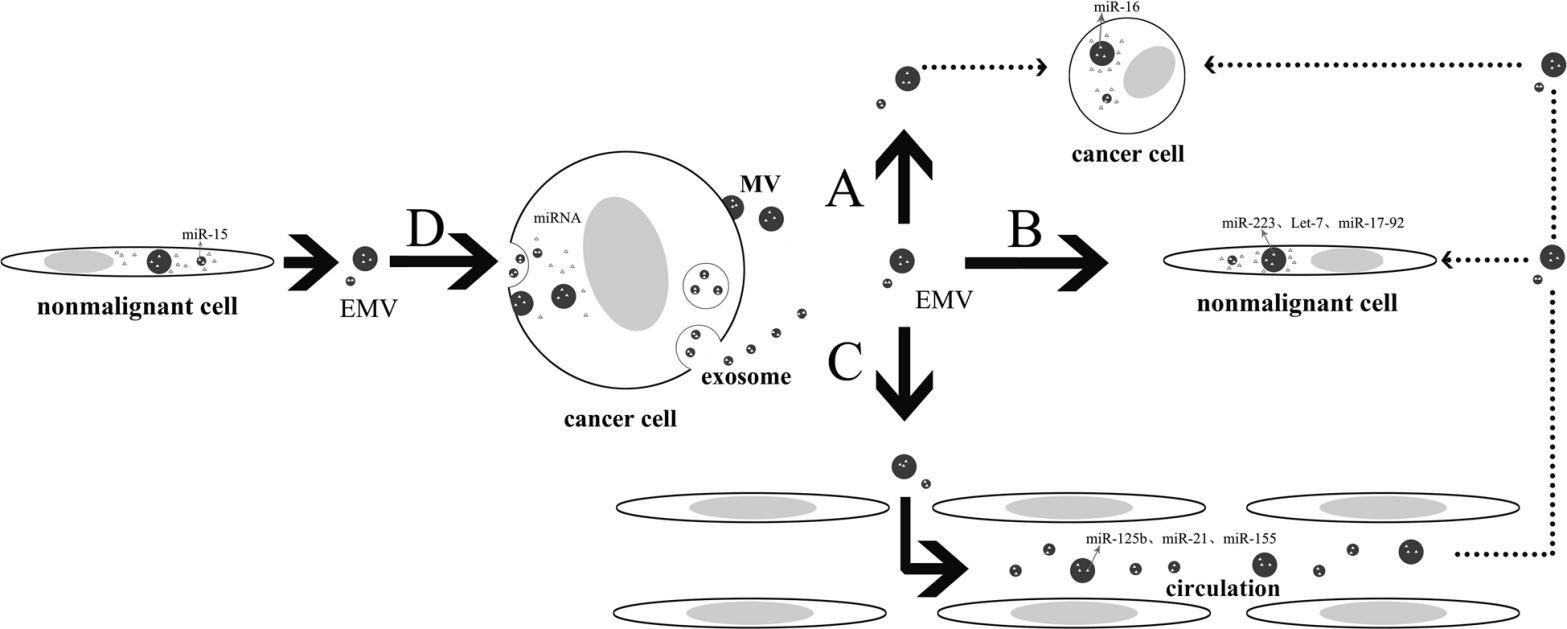
**A new working model of EMV-mediated miRNA transfer in tumor.** As crosstalk mediators, extracellular membrane microvesicles (EMVs), which are derived from cancer cells, carry the selective microRNAs to exert the direct effects into (A) the homological cancer cells to promote malignant transformation; (B) the neighboring cells, such as endothelial cells, to construct the tumor microenvironment; and (C) peripheral circulation to be used as novel diagnostic biomarkers and prognostic monitoring. (D) Meanwhile, cancer cells can also be influenced by absorbing miRNA-containing EMVs from the adjacent nonmalignant cells. MV, microvesicle; miRNA, microRNA.

The characteristics of EMV-mediated miRNA transfer will be summarized in this section. (1) EMVs function as the genetic messengers in intercellular communication but differ from conventional cell-to-cell communication. The circulating miRNAs have been found to be relatively stable in the extracellular milieu and are resistant to plasma ribonucleases (RNase) with a long half-life, even in the unfavorable physiological conditions such as freeze-thawing and extreme pH and room temperature [32-34]. These indicate that the EMV’s lipid bilayers contribute to maintaining the stability of the circulating miRNA to ensure the transfer of their genetic cargo to the recipient cells. (2) As crosstalk mediators, EMVs carry the miRNAs and exert the effects with high specificity. Exosomes derived from the human T-lymphotropic virus type 1-infected cells contain viral mRNA transcripts [35], as well as tumor-derived MVs which were elegantly demonstrated to have oncogene products into the neighboring cells [36]. These miRNAs were selectively enriched into exosomes and selectively released depending on not only the cell types of origin, namely, the miRNA content differing among exosomes derived from regulatory T cell (Treg) and T helper cell type 1 (Th1) and T helper cell type 2 (Th2) cells [37], but also the cell stage (mature dendritic cell (DC) cells versus immature cells) [38], as well as the microenvironment context. The content in exosome derived from DC cells in the cancer microenvironment was found to differ from that in infectious microenvironment [39]. (3) These natural small-sized carriers have the ability to cross biological barriers like the blood–brain barrier [40]. It is worthwhile to note that they all originate from the host with no immunological rejection. This provides a great opportunity to utilize them as potentially effective and safe vehicles for transport genetic elements. (4) As membranous structures, EMVs recycle continuously [41], indicating that EMV-miRNAs are economic, efficient, and crucial mediators in the human body. (5) EMVs, carrying their genetic information, may be internalized by recipient cells which may facilitate their cytoplasmic and nuclear functions [42-44]. As shown in Figure 1, this specific mode of transportation sets EMVs apart from traditional modulators such as cytokines and their receptors.

### EMV-mediated miRNA transfer in pathological conditions

The contribution of EMV-mediated miRNA transfer on various pathological conditions has been addressed, namely, trauma, inflammation, infection, and systematic diseases. The transfer of genetic information from injured cells, confirmed by the altered circulating levels of vascular and inflammation-associated miRNAs in inflammatory patients [45], may explain how the functional and phenotypic changes in stem cells occur without the need of extrinsic factors for transdifferentiation into tissue cells. This evidence opens up the possibility to manipulate tissue repair by the utilization of EMVs carrying specific miRNAs. In addition to hemodynamic disorders like nephritic ischemia, hindlimb ischemia, and myocardial ischemia, EMVs, derived from the mesenchymal cells, can protect organs from acute ischemic injury by delivering their RNA content and contributing to the reprogramming of hypoxic resident parenchymal cells to initiate the regenerative program [46-49]. The studies of EMV-containing immune-related miRNAs suggest that they may have the potential to be a novel mechanism in modulating the immune system [50]. Moreover, they may be a biomarker of autoimmune diseases such as systemic lupus erythematosus (SLE) [51]. Certain patterns of serum miRNAs are believed to be mediated by the EMVs [52]. EMVs may help elucidate the possible pathogenesis of SLE [53].

### Transfer of miRNAs by extracellular membrane microvesicles in the context of tumor pathogenesis, especially the microenvironment

#### EMV-mediated miRNA transfer in tumor pathogenesis

EMV-miRNA transfer plays a crucial role in tumor pathogenesis and may be useful in clinical practice. These aspects can be summarized as four key points. (1) Tumor-derived EMVs can promote malignant transformation via horizontal propagation of selective oncogenes. miRNAs contribute to carcinogenesis not only as oncogenes, (miR-9 detected in EMVs) [54,55], but also as the tumor suppressor genes (miR-21 found in EMVs) [27,56]. (2) The EMV-mediated miRNA transfer determines the fate of the cells by controlling growth or death. Anti-apoptotic miRNAs, the miR-17 cluster, are detected in peripheral blood microvesicles [27] while the serum levels of the homologous series of these miRNAs are usually up regulated in some cancers [57]. (3) EMV-mediated miRNA transfer may drive invasion and metastasis of tumors. At least 30 miRNAs have anti-angiogenic and pro-angiogenic functions in cancers [58]. Yang et al. confirmed that microvesicles shuttle invasion of miRNAs into cancer cells [59]. Two groups of investigators demonstrated that microvesicles modulated the establishment of metastasis [60,61]. (4) EMVs may be used as novel diagnostic biomarkers and in prognostic monitoring due to their stable existence in ubiquitous biological fluids and miRNA panel specific for cancer’s pathophysiological processes. Furthermore, EMVs may be used as potential vehicles to selectively deliver therapeutic nucleic-acid drugs or conventional drugs for tumor therapy. As described in Table 1, in view of this field, the plasma EMV-containing miRNA panel as a sensitive and tumor-specific marker has become a hot topic in the majority of solid tumors, to a large extent, which represents the biological behavior of the tumor itself. However, research on hematological malignancies has focused on the function of EMVs in the pathogenic crosstalk between tumor cells and pericytes, which is expected to make pivotal contributions to the construction of the tumor microenvironment.

**Table 1:** **List of EMV-mediated miRNA transfer in tumor pathogenesis as well as inflammatory/immune pathologies**

**Disease**	**The given name of EMVs**	**EMVs’ derivation**	**EMVs’ target**	**Involved miRNAs**	**Research content**	**References**
*Ex vivo*	Exosome	MC/9 cells	HMC-1 cells MC/9 cells	N/A	Mediating intercellular communication	[26]
Atherosclerosis	MV	THP-1 cells	HMEC-1	miR-150	Enhancing migration	[34]
Inflammation	Exosome	Tregs	Th1 cells	Let-7d	Preventing inflammation	[37]
Kidney ischemia-reperfusion injury	MV	EPCs	Hypoxic resident renal cells	miR-126 miR-296	Protecting resident kidney	[46]
Hindlimb ischemia	MV	EPCs	Endothelial cells	miR-126 miR-296	Pro-neovascularization	[48]
Breast cancer	MV	Macrophages	Cancer cells	miR-223	Promoting invasion	[59]
Gastric cancer	Exosome	AZ-P7a cells	Microenvironment	Let-7	Pro-oncogenesis	[89]
Leukemia	Exosome	K562 cells	HUVECs	miR-17-92	Enhancing migration and tube formation	[90]
MM	Exosome	BMSC	MM cells	miR-15	Facilitating progression	[92]
Glioblastoma multiforme	MV	Serum	Circulating	N/A	Diagnostic marker	[29]
PC	Exosome	PC-3	Conditioned media	N/S	Mediating intercellular communication	[63]
Melanoma	Exosome	Serum	Circulation	miR-125b	Monitor indicator	[67]
HCC	Nano vesicle	Hep3B HepG2 PLC/PRF/5 Cells	Hep3B HepG2 PLC/PRF/5 cells	miR-16	Mediating intercellular communication	[68]
Ovarian cancer	Exosome	Serum	Circulation	N/S	Diagnostic marker	[71]
Breast cancer	Exosome	Serum	Circulation	miR-21	Monitor indicator	[73]
Lung cancer	Exosome	Serum	Circulation	N/S	Diagnostic marker	[74]
NSCLC	Exosome	Serum	Circulation	miR-21 miR-155	Diagnostic marker	[75]

MC/9: Mouse mast cell line; HMC-1: Human mast cell line; THP-1: Human acute monocytic leukemia-1 cell line; HMEC-1: Human dermal microvascular endothelial cell line; PC-3: Prostate cancer cell line; Hep3B, HepG2, PLC/PRF/5: Human HCC cell lines; AZ-P7a cells: Human metastatic duodenal cancer cell lines; K562: Human chronic myeloid leukemia cell line; N/A: Not applicable; N/S: Not specified; MVs: Microvesicles; Treg: regulatory T cell; Th1: T helper cell type 1; EPCs: Endothelial progenitor cells; PC: Prostate cancer; HCC: Hepatocellular carcinoma; HUVECs: Human umbilical vascular endothelial cells; MM: Multiple myeloma; BMSC: Bone marrow stromal cell; NSCLC: Non-small-cell lung cancer; EMVs: extracellular membrane microvesicles.

### EMV-mediated miRNA transfer in solid tumor

EMVs are secreted by many cell types but cancer cells have a higher production of them. Prostate cancer (PC) is the first model which has been profoundly studied in this area [62,63]. Lehmann et al. observed that the senescence of radiation-induced PC cells was associated with a significant increase in the release of EMVs containing a large number of small RNAs (<100 base pairs (bp)) [62]. From the clinical viewpoint, a research team confirmed EMVs ‘as biomarker treasure chests’ for PC diagnosis [64]. One year later, Bryant et al. found that circulating miRNAs, embedded in EMVs, may represent potentially useful biomarkers for the diagnosis, staging, and prognostic prediction of PC [65].

Since the initial studies, serum EMVs were revealed and may be potential biomarkers in other tumors (Table 1) including glioblastoma [29,66], melanoma [67], liver cancer [68,69], gastric cancer [70], ovarian cancer [71,72], breast cancer [73], lung carcinoma [74,75], and Ewing’s sarcoma [76]. However, the roles of EMV-mediated miRNA transfer are still largely unknown and should be studied further in solid tumors.

### EMV-mediated miRNA transfer in hematological malignancies

Etiologically, Yamada et al. confirmed that the bovine leukemia virus proteins were released with milk EMVs and may be transferred into recipient cells of calves via milk EMVs as an alternative route without requiring a virus infection [77]. In chronic myelogenous leukemia, BCR-ABL1-positive EMVs could initiate malignant transformation of normal hematopoietic transplants through genomic instability [78]. Circulating microvesicles during chronic lymphoproliferative diseases were detected and channels regulating leukemia-cell-derived EMV formation were previously discussed [79,80]. Treatment by natural killer cell suppression also involves EMVs [81,82]. Patients who accepted stem cell therapeutic strategies may benefit from the manipulation of paracrine EMVs [83]. Src was shown to play a role in promoting angiogenesis in chronic myeloid leukemia and enrichment of EMVs in malignant plasma cell dyscrasia [84,85]. The effect of EMVs on myeloma has been discussed in the context of tumor cell proliferation and angiogenesis [86-88].

### EMV-mediated miRNA transfer in tumorous angiogenesis

Cancer cells can utilize EMV-mediated miRNA transduction to constitute their microenvironments in stimulating angiogenesis. Tumor EMVs, prostate, breast, and ovarian cancers and some hematological neoplasms, may convey phenotypic transforming signals to non-malignant cells and may acquire tumor-supporting characteristics, namely, vasculogenic and epithelial markers. Zhang et al. showed that human leukemia cell line THP-1 cells selectively packaged miR-150 into multivesicular bodies and actively secreted them into the extracellular environment to enhance target endothelial cell migration [34]. Keiichi Ohshima et al. demonstrated that gastric cancer cells secreted let-7 miRNAs via exosomes into the extracellular environment to maintain the process of oncogenesis [89]. Szczepanski et al. reported that blast-derived microvesicles in sera from patients with acute myeloid leukemia suppress natural killer cell function by membrane-associated transforming growth factor-beta1 [81]. Umezu et al. first circumstantiated that leukemia-endothelial cell communication via exosomal miRNA may, in part, be associated with angiogenic activity in endothelial cells [90]. Recently, Tadokoro showed that exosomes derived from hypoxic leukemia cells enhanced tube formation in endothelial cells [91]. In animal models and human samples, bone marrow mesenchymal stromal cell (BM-MSC)-derived exosomes were reported to have effects on viability, proliferation, survival, migration, and drug resistance of MM cells [92,93]. Luga reported that fibroblast-secreted exosomes promote breast cancer cell protrusive activity and motility by Wnt-planar cell polarity signaling [94]. Moreover, tumor-microenvironment-cell-derived-EMVs may act on other cells types. In 1998, studies on exosomes derived from antigen present cells (APCs) demonstrated that they have the capacity to prime naive CD8+ T lymphocytes to eradicate tumors [95]. Gastpar showed for the first time that exosomes originating from Hsp70/Bag-4 membrane-positive tumor cells stimulated the migration and reactivity of Hsp70 in natural killer (NK) cells [96]. Cell communication via EMVs is complicated but fascinating and participates not only in normal physiology but also in pathological phenomena, namely, cancer. Research studies have found a variety of functions of EMVs and further attention needs to be focused in this area.

## Summary

EMVs are novel and unique effectors carrying out many biological messages. Aside from their conventional basal intracellular communications, EMVs can also actively participate in triggering signal pathways and exclusively transfer nucleic acids as miRNA clusters. Due to its characteristics, microvesicle-mediated miRNA transduction is one of the first autogenous mediators to exchange specific and endogenous tumor-related genetic signals among multiple types of cells, which has attracted more attention on malignant cell-endothelial cell interaction in the tumor microenvironment. Therapy by microvesicle-mediated miRNA transduction may aid in drug resistance and inhibition of tumor invasion. Collectively, these studies suggest that this novel intracellular model complements the classic cell-to-cell communication and provides new therapeutic strategies for both solid cancers and hematological disorders.

## Abbreviations

APCs: antigen present cells
BM-MSCs: bone marrow mesenchymal stromal cells
bp: base pair
DCs: dendritic cells
DNA: deoxyribo nucleic acid
EMVs: extracellular membrane microvesicles
miRNAs: microRNAs
MM: multiple myeloma
mRNA: message ribose nucleic acid
NCI: National Cancer Institute
NK: natural killer
PC: prostate cancer
RNase: ribonucleases
SLE: systemic lupus erythematosus
Th1: T helper cell type 1
Th2: T helper cell type 2
Treg: regulatory T cell
μm: micrometer
